# Crystal structure of bis­{5-(4-chloro­phen­yl)-3-[6-(1*H*-pyrazol-1-yl)pyridin-2-yl]-1*H*-1,2,4-triazol-1-ido}nickel(II) methanol disolvate

**DOI:** 10.1107/S2056989024010338

**Published:** 2024-10-31

**Authors:** Kateryna Znovjyak, Sergiu Shova, Dmitriy M. Panov, Nataliia S. Kariaka, Igor O. Fritsky, Sergey O. Malinkin, Maksym Seredyuk

**Affiliations:** a Department of Chemistry, Taras Shevchenko National University of Kyiv, Volodymyrska Street 64, Kyiv, 01601, Ukraine; b Department of Inorganic Polymers "Petru Poni" Institute of Macromolecular Chemistry Romanian Academy of Science Aleea Grigore Ghica Voda 41-A Iasi 700487 Romania; c ChemBioCenter Kyiv National Taras Shevchenko University Kyiv 02094 61 Winston Churchill Street Ukraine; University of Neuchâtel, Switzerland

**Keywords:** crystal structure, nickel(II) complexes, neutral complexes, tridentate ligands, bis­azole­pyridines

## Abstract

The unit cell of the title compound, [Ni(C_16_H_10_ClN_6_)_2_]·2CH_3_OH, consists of a neutral complex and two methanol mol­ecules. In the complex, the two tridentate 2-(3-(4-chloro­phen­yl)-1*H*-1,2,4-triazol-5-yl)-6-(1*H*-pyrazol-1-yl)pyridine ligands coordinate to the central Ni^II^ ion through the N atoms of the pyrazole, pyridine and triazole groups, forming a pseudo­octa­hedral coordination sphere. Neighbouring tapered mol­ecules are linked through weak C—H(pz)⋯π(ph) inter­actions into monoperiodic chains, which are further linked through weak C—H⋯N/C inter­actions into diperiodic layers. The inter­molecular contacts were qu­anti­fied using Hirshfeld surface analysis and two-dimensional fingerprint plots, revealing the relative contributions of the contacts to the crystal packing to be H⋯H 32.8%, C⋯H/H⋯C 27.5%, N⋯H/H⋯N 15.1%, and Cl⋯H/H⋯Cl 14.0%. The average Ni—N bond distance is 2.095 Å. Energy framework analysis at the HF/3–21 G theory level was performed to qu­antify the inter­action energies in the crystal structure.

## Abstract 2

The title compound, a neutral bis­{5-(4-chloro­phen­yl)-3-[6-(1*H*-pyrazol-1-yl)pyridin-2-yl]-1*H*-1,2,4-triazol-1-ido}nickel(II) methanol disolvate has a distorted pseudo­octa­hedral coordination environment of the metal ion. As a result of the tapered shape and polar nature, the mol­ecules stack in one-dimensional columns that are bound by weak hydrogen bonds into layers, which are arranged in a three-dimensional structure without inter­layer inter­actions below van der Waals radii.

## 1. Chemical context

A broad class of coordination compounds is represented by 3*d*-metal complexes based on tridentate bis­azole­pyridine ligands (Halcrow *et al.*, 2019 ▸; Suryadevara *et al.*, 2022 ▸), which find application in many fields, for example in catalysis (Xing *et al.*, 2014 ▸; Wei *et al.*, 2015 ▸) and mol­ecular magnetism (Suryadevara *et al.*, 2022 ▸). In the case of asymmetric ligand design, where one of the azole groups carries a hydrogen on a nitro­gen heteroatom and acts as a Brønsted acid, deprotonation can compensate for the charge of the central ion and in some cases form neutral complexes (Seredyuk *et al.*, 2014 ▸; Grunwald *et al.*, 2023 ▸). The periphery of the complexes also plays an important role, determining the way the mol­ecules inter­act with each other, influencing the inter­molecular connectivity, inter­action energy and the organization of the crystal.

Encouraged by our inter­est in spin-transition complexes of 3*d*-metals formed by N-heterocyclic ligands (Seredyuk *et al.*, 2006 ▸, 2007 ▸; Bonhommeau *et al.*, 2012 ▸; Piñeiro-López *et al.*, 2018 ▸), we report a new neutral Ni^II^ complex based on an asymmetric deprotonated ligand with a monosubstituted phenyl group, 2-{5-[5-(4-chloro­phen­yl)-1*H*-1,2,4-triazol-3-yl]-6-(1*H*-pyrazol-1-yl)pyridine}, which continues our enduring project on the study of metal complexes of bis­azole­pyridines.
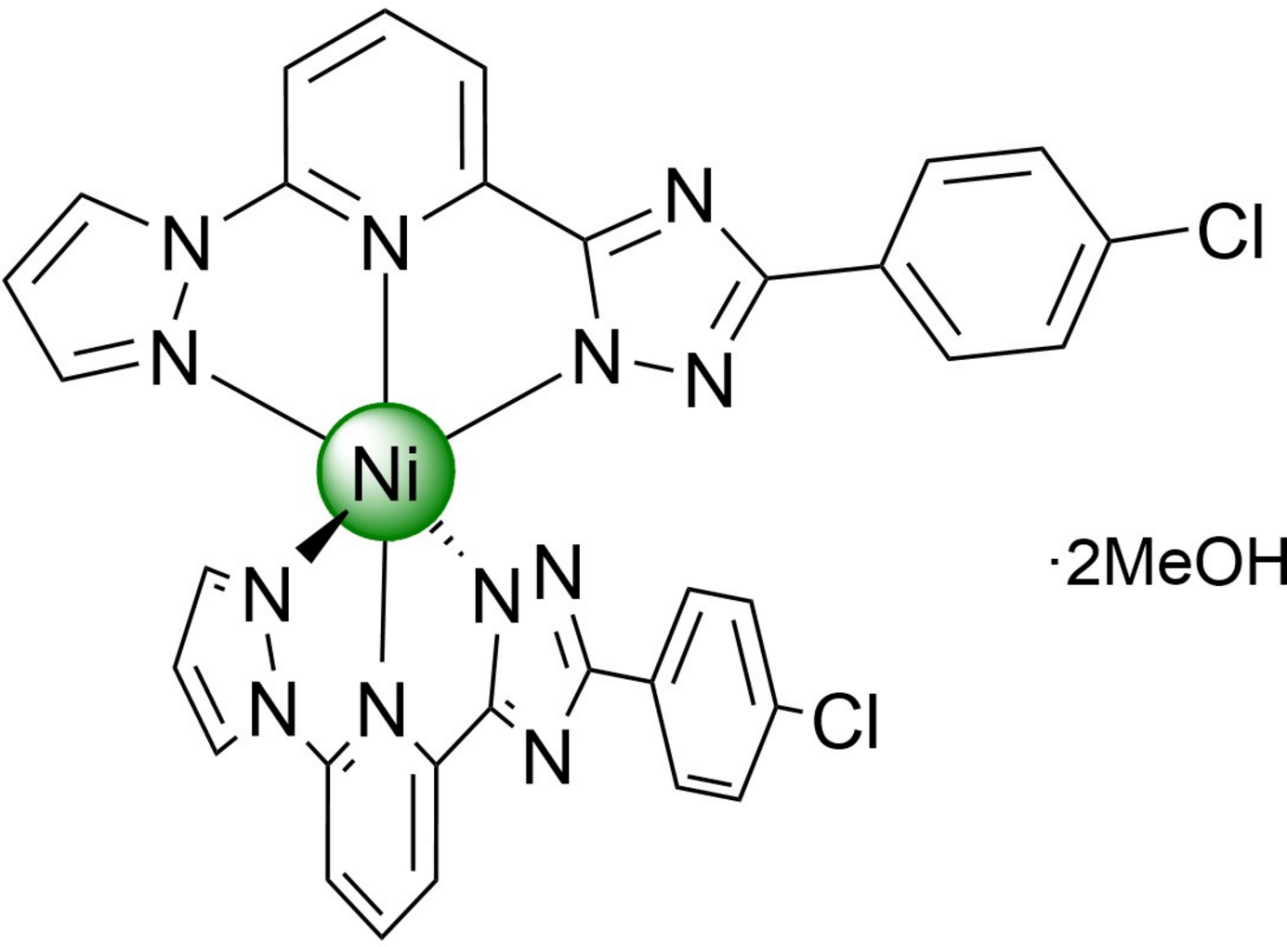


## 2. Structural commentary

**Figure 1:**
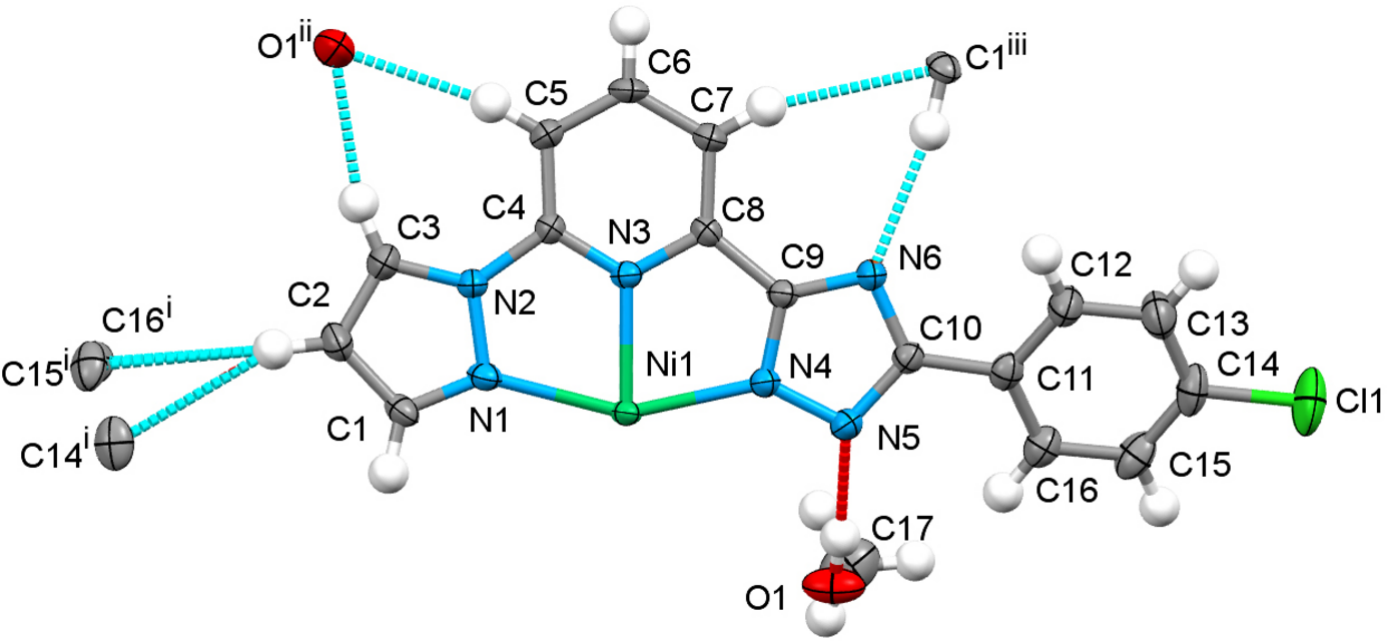
The mol­ecular structure of half of the title compound, with displacement ellipsoids drawn at the 50% probability level. The strong O—H⋯N (red) and weak C–H⋯N/C/O (cyan) hydrogen bonds are shown with the nearest neighbours. Symmetry codes: (i) 1 − *x*, 1 + *y*, 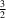 − *z*; (ii) −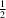 + *x*, 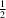 + *y*, 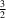 − *z*; (iii) 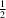 + *x*, 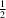 + *y*, 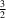 − *z*.

The complex has a tapered structure with divergent phenyl groups. The phenyl group of the ligand is rotated by 26.2 (1)° relative to the pyrazole-pyridine-triazole (pz-py-trz) fragment, the arrangement of which is almost planar. There are two methanol mol­ecules per complex, forming O—H⋯N hydrogen bonds with the trz rings (Fig. 1 ▸, Table 1 ▸). The central Ni ion has a distorted octa­hedral N_6_ coordination environment formed by the nitro­gen donor atoms of two tridentate ligands. The average Ni—N bond length is 2.095 Å. The average trigonal distortion parameters *Σ* = Σ_1_^12^(|90 − *φ*_i_|), where *φ*_i_ is the angle N—Ni—N′ (Drew *et al.*, 1995 ▸), and *Θ* = Σ_1_^24^(|60 − *θ*_i_|), where *θ*_i_ is the angle generated by superposition of two opposite faces of an octa­hedron (Chang *et al.*, 1990 ▸) are 119.4 and 387.3°, respectively. The values reveal a deviation of the coordination environment from an ideal octa­hedron (where *Σ* = *Θ* = 0), which is, however, in the expected range for bis­azole­pyridine and similar ligands (see below). The calculated continuous shape measures [CShM(O_*h*_)] value relative to the ideal octa­hedral symmetry is 3.714 (Kershaw Cook *et al.*, 2015 ▸). The volume of the [NiN_6_] coordination polyhedron is 11.583 Å^3^.

## 3. Supra­molecular features

**Figure 2:**
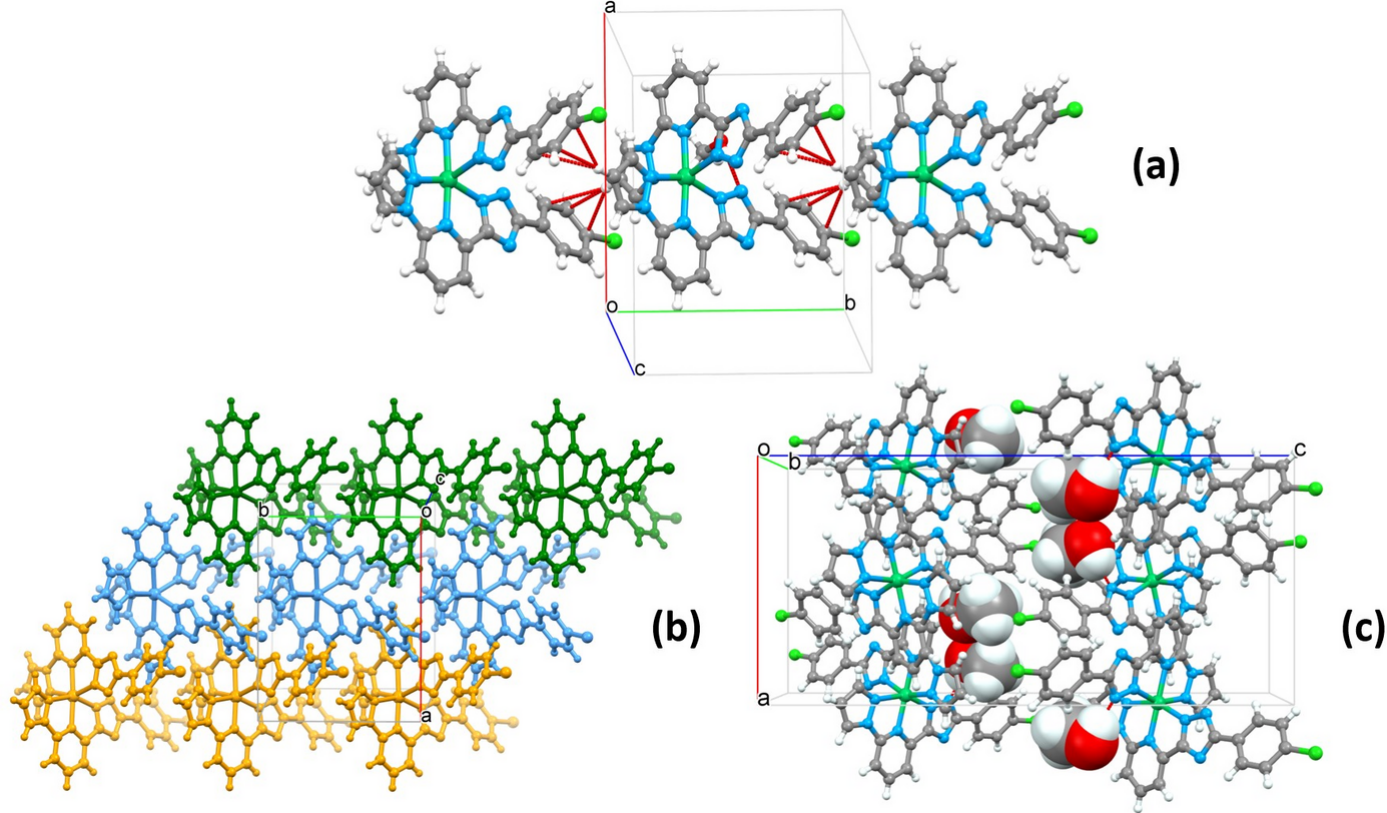
(*a*) A fragment of the monoperiodic supra­molecular column formed by stacking of mol­ecules along the *b* axis; (*b*) supra­molecular diperiodic layers formed by stacking of the supra­molecular columns in the *ac* plane. For a better representation, each column has a different colour; (*c*) stacking of the diperiodic layers along the *b* axis with the methanol mol­ecules (CPK model) in the voids.

As a result of the tapered structure, neighbouring complexes are embedded in each other and inter­act through weak C–H(pz)⋯π(ph) inter­molecular contacts between the pyrazole and phenyl groups [the C2⋯*C_g_*(ph) distance is 3.534 Å]. They form one-dimensional supra­molecular chains extending along the *b-*axis direction with a stacking periodicity equal to 10.1523 (4) Å (= cell parameter *b*) (Fig. 2 ▸)*.* Weak inter­molecular C—H(pz, py)⋯N/C(pz, trz) inter­actions, ranging from 3.245 (4) to 3.743 (4) Å (Table 1 ▸), connect neighbouring chains into two-dimensional layers along the *ab* plane. The voids between the layers are occupied by solvent mol­ecules, which also participate in the bonding within separate layers. The methanol mol­ecules form a strong O—H⋯N hydrogen bond with the deprotonated trz groups and weak hydrogen bonds C—H⋯O with the pz and py groups of the ligand. A complete list of selected inter­molecular inter­actions is provided in Table 1 ▸.

## 4. Hirshfeld surface and two-dimensional fingerprint plots

**Figure 3:**
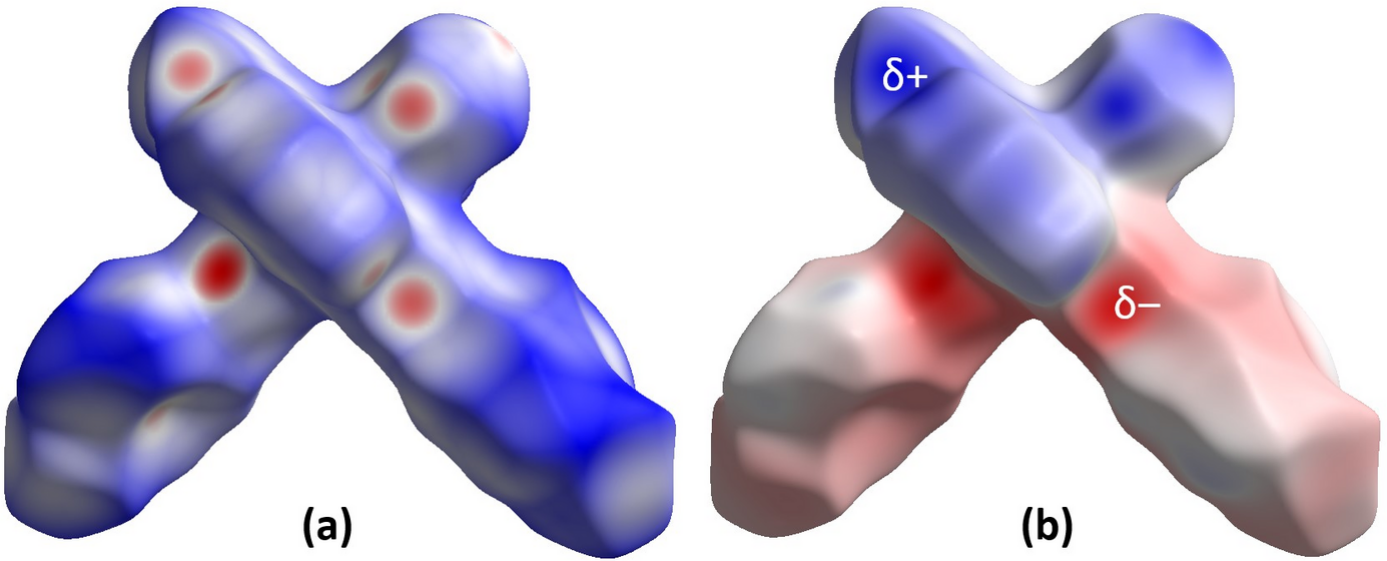
(*a*) A projection of *d*_norm_ mapped over the Hirshfeld surfaces, showing the inter­molecular inter­actions within the mol­ecule. Red/blue and white areas represent regions where contacts are shorter/larger than the sum and close to the sum of the van der Waals radii, respectively; (*b*) electrostatic potential for the title compound mapped over the Hirshfeld surface. Red/blue and white areas represent regions where the charge is negative/positive or close to zero.

**Figure 4:**
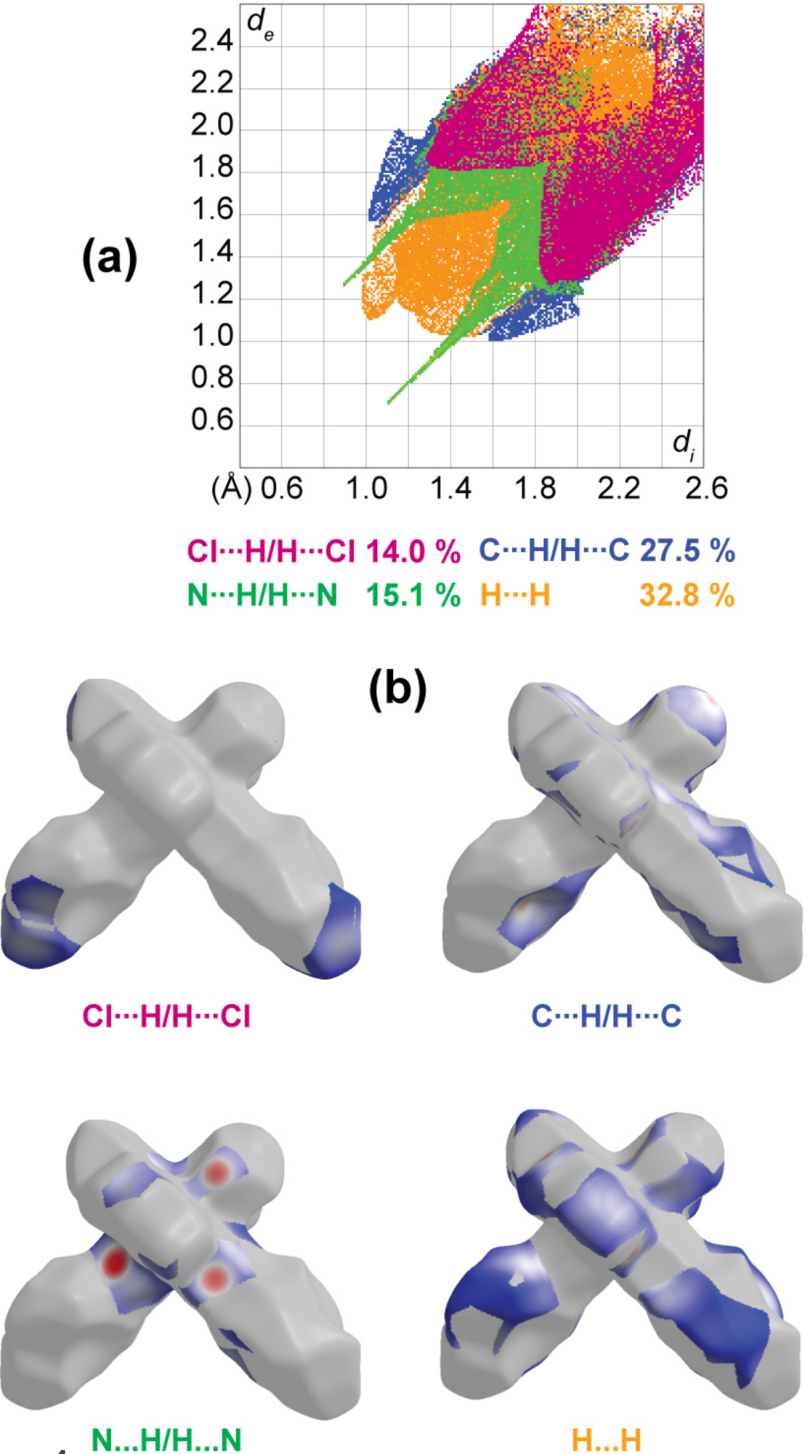
(*a*) Decomposition of the two-dimensional fingerprint plot into specific inter­actions; (*b*) a projection of *d*_norm_ mapped on the Hirshfeld surfaces, showing the inter­molecular inter­actions within the mol­ecule. Red/blue and white areas represent regions where contacts are shorter/larger than the sum and close to the sum of the van der Waals radii, respectively.

Hirshfeld surface analysis was performed and the associated two-dimensional fingerprint plots were generated using *CrystalExplorer* (Spackman *et al.*, 2021 ▸), with a standard resolution of the three-dimensional *d*_norm_ surfaces plotted over a fixed colour scale from −0.6356 (red) to 1.6114 (blue) a.u. The pale-red spots symbolize short contacts and negative *d*_norm_ values on the surface corresponding to the inter­actions described above. The overall two-dimensional fingerprint plot is illustrated in Fig. 3 ▸*a*. The two-dimensional fingerprint plots, with their relative contributions to the Hirshfeld surface mapped over *d*_norm_, are shown for the H⋯H, C⋯H/H⋯C, N⋯H/H⋯N and Cl⋯H/H⋯Cl contacts in Fig. 4 ▸. At 32.8%, the largest contribution to the overall crystal packing is from H⋯H inter­actions, which are located in the middle region of the fingerprint plot. C⋯H/H⋯C contacts contribute 27.5%, and Cl⋯H/H⋯Cl 14.0%, resulting in pairs of characteristic wings. The N⋯H/H⋯N contacts, represented by a pair of sharp spikes in the fingerprint plot, make a 15.1% contribution to the surface. The electrostatic potential energy calculated using the HF/3-21G basis is mapped on the Hirshfeld surface (Fig. 3 ▸*b*). The negative charge localizes on the trz-ph moiety and the Cl atom of the complex, while the pz-py moiety is relatively positively charged, which justifies the stacking of the mol­ecules in columns and packing of the columns in diperiodic two-dimensional layers.

## 5. Energy frameworks analysis

**Figure 5:**
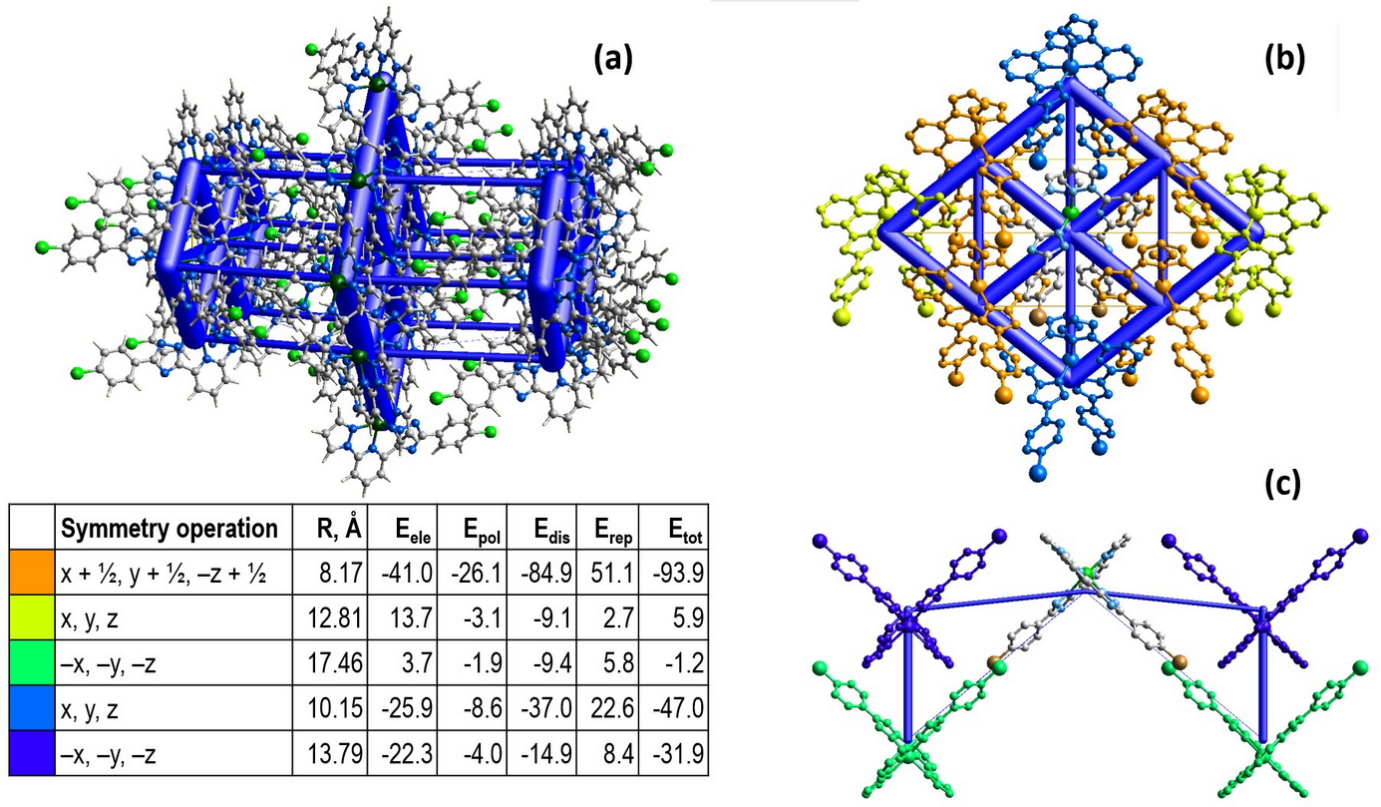
(*a*) The calculated energy frameworks, showing the total energy diagrams (*E*_tot_), (*b*) decomposition of the energy framework into the part corresponding to the inter­actions within a supra­molecular layer and (*c*) inter­layer inter­actions. In the table the corresponding colour-coded energy values *E*_tot_ are provided, including their *E*_ele_, *E*_pol_, *E*_dis_, and *E*_rep_ components. Tube size is set at 100 scale, the blue colour corresponds to the attractive inter­action, yellow to the repulsive inter­actions.

The energy framework (Spackman *et al.*, 2021 ▸), calculated using the wave function at the HF/3-21G theory level, including the electrostatic potential forces (*E*_ele_), the dispersion forces (*E*_dis_) and the total energy diagrams (*E*_tot_), is shown in Fig. 5 ▸. The cylindrical radii, adjusted to the same scale factor of 100, are proportional to the relative strength of the corresponding energies. The major contribution is due to the dispersion forces (*E*_dis_), reflecting dominating inter­actions in the crystal of the neutral mol­ecules. The topology of the energy framework resembles the topology of the inter­actions within and between layers described above. The calculated value *E*_tot_ for the intra­chain inter­actions is −47.0 kJ mol^−1^ and for inter­chain inter­actions is down to −93.9 kJ mol^−1^. The inter­layer inter­actions have an energy of −31.9 kJ mol^−1^. The colour-coded inter­action mappings within a radius of 3.8 Å from the complex, together with full information on the various contributions to the total energy (*E*_ele_, *E*_pol_, *E*_dis_, *E*_rep_) are shown in the table in Fig. 5 ▸.

## 6. Database survey

A search of the Cambridge Structural Database (CSD, Version 5.42; Groom *et al.*, 2016 ▸) reveals several similar neutral 3*d M*^II^ complexes with tridentate bis­azolepyridine ligands with a deprotonated azole group, for example, of Ni^II^: YOCFAZ (Yuan *et al.*, 2014 ▸), ZOCKOT (Xing *et al.*, 2014 ▸), and ZOTVIP (Wei *et al.*, 2015 ▸); of Fe^II^: EGIDIL (Seredyuk *et al.*, 2024 ▸), LUTGEO (Senthil Kumar *et al.*, 2015 ▸), and XODCEB (Shiga *et al.*, 2019 ▸). In addition, two related complexes based on phenanthroline-benzimidazole, DOMQUT (Seredyuk *et al.*, 2014 ▸) and di­pyridyl­pyrrol, NIRLOT (Grunwald *et al.*, 2023 ▸) were found. The values of the trigonal distortion indices and the CShM(O_*h*_) values vary according to the length of the *M*—N distances, with shorter distances being systematically smaller. Table 2 ▸ collates the structural parameters of the complexes and of the title compound.

## 7. Synthesis and crystallization

The synthesis of the title compound was identical to that reported for a similar complex (Seredyuk *et al.*, 2022 ▸). It was produced by using a layering technique in a standard test tube. The layering sequence was as follows: the bottom layer contained a solution of [Ni(*L*_2_)](ClO_4_)_2_ prepared by dissolv­ing *L* = 2-[3-(4-chloro­phen­yl)-1*H*-1,2,4-triazol-5-yl]-6-(1*H*-pyra­zol-1-yl)pyridine (89 mg, 0.274 mmol) and Ni(ClO_4_)_2_·6H_2_O (50 mg, 0.137 mmol) in boiling acetone, to which chloro­form (5 ml) was then added. The middle layer was a methanol–chloro­form mixture (1:10) (10 ml), which was covered by a layer of methanol (10 ml), to which 100 ml of NEt_3_ were added dropwise. The tube was sealed, and violet plate-like single crystals appeared after 2 weeks (yield *ca* 65%). Elemental analysis calculated for C_34_H_28_Cl_2_N_12_NiO_2_: C, 53.29; H, 3.68; N, 21.94. Found: C, 53.64; H, 3.42; N, 21.67.

## 8. Refinement

Crystal data, data collection and structure refinement details are summarized in Table 3 ▸. H atoms were refined as riding [C—H = 0.95–0.98 Å with *U*_iso_(H) = 1.2–1.5*U*_eq_(C)], while the O-bound H atom was refined freely with *U*_iso_(H) = 1.5*U*_eq_(O).

## Supplementary Material

Crystal structure: contains datablock(s) I. DOI: 10.1107/S2056989024010338/tx2090sup1.cif

Structure factors: contains datablock(s) I. DOI: 10.1107/S2056989024010338/tx2090Isup2.hkl

Supporting information file. DOI: 10.1107/S2056989024010338/tx2090Isup3.cdx

Supporting information file. DOI: 10.1107/S2056989024010338/tx2090Isup4.cdx

Supporting information file. DOI: 10.1107/S2056989024010338/tx2090Isup5.cdx

CCDC reference: 2393088

Additional supporting information:  crystallographic information; 3D view; checkCIF report

## Figures and Tables

**Table 1:** Hydrogen-bond geometry (Å, °)

*D*—H⋯*A*	*D*—H	H⋯*A*	*D*⋯*A*	*D*—H⋯*A*
C2—H2⋯C14^i^	0.95	2.86	3.73 (4)	153
C2—H2⋯C15^i^	0.95	2.74	3.686 (4)	178
C2—H2⋯C16^i^	0.95	2.88	3.743 (4)	151
C3—H3⋯O1^ii^	0.95	2.35	3.259 (4)	160
C5—H5⋯O1^ii^	0.95	2.47	3.399 (4)	167
C1—H1⋯N6^iii^	0.95	2.31	3.245 (6)	170
C7—H7⋯C1^iii^	0.95	2.70	3.611 (4)	161
O1—H1*A*⋯N5	0.84	1.96	2.795 (6)	176

Symmetry codes: (i) 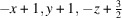; (ii) 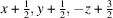; (iii) 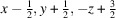.

**Table 2:** Computed distortion indices (Å, °) for the title compound and for similar complexes reported in the literature

CSD Refcode	Metal ion	<*M*—*N*>^*a*^	*Σ*	*Θ*	CShM(O_h_)
Title compound	Ni	2.095	119.4	387.3	3.71
YOCFAZ	Ni	2.088^*b*^	120.8^*b*^	397.6^*b*^	3.65^*b*^
ZOCKOT	Ni	2.086	121.0	375.9	3.78
ZOTVIP	Ni	2.110	124.9	382.4	3.55
EGIDIL	Fe	1.955	89.8	314.6	2.25
EGIDIL02	Fe	2.167	146.8	492.8	5.28
LUTGEO	Fe	1.933	85.0	309.6	2.10
XODCEB	Fe	1.950	87.4	276.6	1.93
DOMQUT	Fe	1.991	88.5	320.0	2.48
DOMQUT02	Fe	2.183	139.6	486.9	5.31
NIRLOT	Fe	1.939	77.3	255.6	1.68

Notes: (*a*) averaged value; (*b*) value is averaged for two independent mol­ecules.

**Table 3:** Experimental details

Crystal data	
Chemical formula	[Ni(C_16_H_10_ClN_6_)_2_]·2CH_4_O
*M*_r_	766.29
Crystal system, space group	Orthorhombic, *Pbcn*
Temperature (K)	200
*a*, *b*, *c* (Å)	12.8146 (4), 10.1523 (4), 27.5618 (10)
*V* (Å^3^)	3585.7 (2)
*Z*	4
Radiation type	Mo *K*α
μ (mm^−1^)	0.74
Crystal size (mm)	0.3 × 0.2 × 0.03

Data collection	
Diffractometer	Xcalibur, Eos
Absorption correction	Multi-scan (*CrysAlis PRO*; Rigaku OD, 2024 ▸)
*T*_min_, *T*_max_	0.982, 1.000
No. of measured, independent and observed [*I* > 2σ(*I*)] reflections	11432, 3169, 2460
*R*_int_	0.040
(sin θ/λ)_max_ (Å^−1^)	0.595

Refinement	
*R*[*F*^2^ > 2σ(*F*^2^)], *wR*(*F*^2^), *S*	0.042, 0.087, 1.05
No. of reflections	3169
No. of parameters	236
H-atom treatment	H atoms treated by a mixture of independent and constrained refinement
Δρ_max_, Δρ_min_ (e Å^−3^)	0.38, −0.43

Computer programs: *CrysAlis PRO* (Rigaku OD, 2024 ▸), *SHELXL2018/3* (Sheldrick, 2015 ▸) and *OLEX2* (Dolomanov *et al.*, 2009 ▸).

## supplementary crystallographic information

### Bis{5-(4-chlorophenyl)-3-[6-(1H-pyrazol-1-yl)pyridin-2-yl]-1H-1,2,4-triazol-1-ido}nickel(II) methanol disolvate Crystal data

**Table:**

[Ni(C_16_H_10_ClN_6_)_2_]·2CH_4_O	*D*_x_ = 1.419 Mg m^−^^3^
*M**_r_* = 766.29	Mo *K*α radiation, λ = 0.71073 Å
Orthorhombic, *P**b**c**n*	Cell parameters from 3630 reflections
*a* = 12.8146 (4) Å	θ = 2.2–26.9°
*b* = 10.1523 (4) Å	µ = 0.74 mm^−^^1^
*c* = 27.5618 (10) Å	*T* = 200 K
*V* = 3585.7 (2) Å^3^	Plate, clear light colourless
*Z* = 4	0.3 × 0.2 × 0.03 mm
*F*(000) = 1576	

### Bis{5-(4-chlorophenyl)-3-[6-(1H-pyrazol-1-yl)pyridin-2-yl]-1H-1,2,4-triazol-1-ido}nickel(II) methanol disolvate Data collection

**Table:**

Xcalibur, Eos diffractometer	3169 independent reflections
Radiation source: fine-focus sealed X-ray tube, Enhance (Mo) X-ray Source	2460 reflections with *I* > 2σ(*I*)
Graphite monochromator	*R*_int_ = 0.040
Detector resolution: 16.1593 pixels mm^-1^	θ_max_ = 25.0°, θ_min_ = 2.2°
ω scans	*h* = −15→12
Absorption correction: multi-scan (CrysAlisPro; Rigaku OD, 2024)	*k* = −8→12
*T*_min_ = 0.982, *T*_max_ = 1.000	*l* = −28→32
11432 measured reflections	

### Bis{5-(4-chlorophenyl)-3-[6-(1H-pyrazol-1-yl)pyridin-2-yl]-1H-1,2,4-triazol-1-ido}nickel(II) methanol disolvate Refinement

**Table:**

Refinement on *F*^2^	0 restraints
Least-squares matrix: full	Hydrogen site location: mixed
*R*[*F*^2^ > 2σ(*F*^2^)] = 0.042	H atoms treated by a mixture of independent and constrained refinement
*wR*(*F*^2^) = 0.087	*w* = 1/[σ^2^(*F*_o_^2^) + (0.0279*P*)^2^ + 2.4046*P*] where *P* = (*F*_o_^2^ + 2*F*_c_^2^)/3
*S* = 1.05	(Δ/σ)_max_ < 0.001
3169 reflections	Δρ_max_ = 0.38 e Å^−^^3^
236 parameters	Δρ_min_ = −0.43 e Å^−^^3^

### Bis{5-(4-chlorophenyl)-3-[6-(1H-pyrazol-1-yl)pyridin-2-yl]-1H-1,2,4-triazol-1-ido}nickel(II) methanol disolvate Special details

**Table:**

Geometry. All esds (except the esd in the dihedral angle between two l.s. planes) are estimated using the full covariance matrix. The cell esds are taken into account individually in the estimation of esds in distances, angles and torsion angles; correlations between esds in cell parameters are only used when they are defined by crystal symmetry. An approximate (isotropic) treatment of cell esds is used for estimating esds involving l.s. planes.

### Bis{5-(4-chlorophenyl)-3-[6-(1H-pyrazol-1-yl)pyridin-2-yl]-1H-1,2,4-triazol-1-ido}nickel(II) methanol disolvate Fractional atomic coordinates and isotropic or equivalent isotropic displacement parameters (Å^2^)

**Table:**

	*x*	*y*	*z*	*U*_iso_*/*U*_eq_	
Ni1	0.500000	0.69121 (4)	0.750000	0.02084 (14)	
Cl1	0.65525 (9)	0.02490 (10)	0.48765 (3)	0.0732 (3)	
N4	0.56268 (15)	0.5528 (2)	0.70190 (7)	0.0232 (5)	
N3	0.65534 (14)	0.6994 (2)	0.76563 (7)	0.0213 (5)	
N6	0.70584 (15)	0.4439 (2)	0.67758 (7)	0.0260 (5)	
O1	0.35256 (17)	0.5580 (2)	0.61839 (9)	0.0536 (7)	
N1	0.51184 (15)	0.8366 (2)	0.80671 (8)	0.0254 (5)	
N2	0.61374 (15)	0.8598 (2)	0.82014 (8)	0.0248 (5)	
N5	0.53272 (16)	0.4702 (2)	0.66553 (8)	0.0261 (5)	
C9	0.66590 (18)	0.5348 (2)	0.70733 (9)	0.0225 (6)	
C11	0.6257 (2)	0.3115 (3)	0.61194 (9)	0.0286 (6)	
C10	0.62050 (19)	0.4072 (2)	0.65185 (9)	0.0240 (6)	
C4	0.69229 (18)	0.7853 (2)	0.79721 (9)	0.0226 (6)	
C8	0.72106 (18)	0.6191 (2)	0.74160 (9)	0.0232 (6)	
C1	0.4560 (2)	0.9114 (3)	0.83634 (9)	0.0278 (6)	
H1	0.381914	0.915928	0.836126	0.033*	
C5	0.79770 (19)	0.8008 (3)	0.80689 (10)	0.0308 (7)	
H5	0.822378	0.864474	0.829382	0.037*	
C12	0.7100 (2)	0.2265 (3)	0.60884 (10)	0.0387 (7)	
H12	0.762240	0.228747	0.633302	0.046*	
C15	0.5598 (3)	0.2190 (3)	0.53748 (10)	0.0409 (8)	
H15	0.508238	0.216511	0.512739	0.049*	
C16	0.5500 (2)	0.3065 (3)	0.57582 (10)	0.0346 (7)	
H16	0.491181	0.363367	0.577433	0.041*	
C14	0.6440 (3)	0.1360 (3)	0.53531 (10)	0.0410 (8)	
C3	0.6197 (2)	0.9480 (3)	0.85679 (10)	0.0341 (7)	
H3	0.681449	0.979665	0.871858	0.041*	
C7	0.82769 (19)	0.6262 (3)	0.74938 (10)	0.0316 (7)	
H7	0.874286	0.569090	0.732760	0.038*	
C2	0.5197 (2)	0.9827 (3)	0.86793 (11)	0.0373 (7)	
H2	0.497898	1.043138	0.892220	0.045*	
C13	0.7197 (3)	0.1382 (3)	0.57077 (11)	0.0464 (8)	
H13	0.777684	0.080013	0.569161	0.056*	
C6	0.8646 (2)	0.7190 (3)	0.78215 (10)	0.0352 (7)	
H6	0.937557	0.726096	0.787585	0.042*	
C17	0.3810 (3)	0.6355 (4)	0.57866 (13)	0.0666 (11)	
H17A	0.401889	0.578408	0.551683	0.100*	
H17B	0.439474	0.692602	0.587693	0.100*	
H17C	0.321473	0.689799	0.568711	0.100*	
H1A	0.407 (3)	0.529 (3)	0.6315 (12)	0.062 (11)*	

### Bis{5-(4-chlorophenyl)-3-[6-(1H-pyrazol-1-yl)pyridin-2-yl]-1H-1,2,4-triazol-1-ido}nickel(II) methanol disolvate Atomic displacement parameters (Å^2^)

**Table:**

	*U*^11^	*U*^22^	*U*^33^	*U*^12^	*U*^13^	*U*^23^
Ni1	0.0141 (2)	0.0264 (3)	0.0220 (3)	0.000	−0.00045 (19)	0.000
Cl1	0.1083 (8)	0.0673 (7)	0.0441 (6)	0.0039 (6)	0.0019 (5)	−0.0299 (5)
N4	0.0192 (11)	0.0277 (12)	0.0226 (12)	−0.0017 (9)	−0.0003 (9)	−0.0023 (10)
N3	0.0172 (10)	0.0247 (12)	0.0219 (12)	−0.0013 (9)	−0.0013 (8)	0.0004 (10)
N6	0.0214 (11)	0.0309 (13)	0.0257 (12)	0.0007 (9)	−0.0002 (9)	−0.0073 (10)
O1	0.0324 (13)	0.0715 (17)	0.0569 (16)	−0.0044 (12)	−0.0083 (11)	0.0242 (13)
N1	0.0172 (11)	0.0318 (13)	0.0273 (12)	0.0025 (9)	−0.0012 (9)	0.0001 (10)
N2	0.0190 (11)	0.0281 (12)	0.0273 (12)	0.0009 (9)	−0.0016 (9)	−0.0066 (11)
N5	0.0234 (12)	0.0288 (13)	0.0261 (13)	−0.0031 (9)	−0.0016 (9)	−0.0038 (11)
C9	0.0182 (13)	0.0261 (15)	0.0231 (14)	−0.0005 (10)	0.0000 (11)	−0.0007 (12)
C11	0.0332 (15)	0.0261 (15)	0.0264 (15)	−0.0059 (12)	0.0032 (12)	−0.0007 (13)
C10	0.0248 (14)	0.0249 (14)	0.0223 (14)	−0.0023 (11)	0.0000 (11)	−0.0011 (12)
C4	0.0211 (13)	0.0236 (14)	0.0230 (14)	−0.0006 (11)	−0.0002 (11)	−0.0034 (12)
C8	0.0204 (13)	0.0252 (14)	0.0239 (14)	0.0014 (11)	0.0032 (11)	−0.0013 (12)
C1	0.0218 (13)	0.0344 (16)	0.0271 (16)	0.0081 (12)	0.0020 (12)	0.0005 (13)
C5	0.0222 (14)	0.0355 (16)	0.0347 (16)	−0.0032 (12)	−0.0032 (12)	−0.0118 (14)
C12	0.0413 (18)	0.0392 (18)	0.0357 (17)	0.0035 (14)	−0.0053 (14)	−0.0104 (15)
C15	0.058 (2)	0.0399 (19)	0.0250 (16)	−0.0099 (16)	−0.0079 (14)	0.0027 (15)
C16	0.0413 (17)	0.0325 (17)	0.0299 (16)	−0.0039 (14)	−0.0024 (13)	−0.0007 (14)
C14	0.064 (2)	0.0343 (18)	0.0248 (16)	−0.0042 (16)	0.0065 (15)	−0.0068 (14)
C3	0.0307 (15)	0.0366 (17)	0.0351 (17)	−0.0001 (13)	−0.0034 (13)	−0.0123 (15)
C7	0.0181 (13)	0.0383 (16)	0.0385 (17)	0.0034 (11)	−0.0015 (12)	−0.0111 (15)
C2	0.0333 (17)	0.0425 (18)	0.0360 (17)	0.0097 (13)	0.0023 (13)	−0.0128 (15)
C13	0.052 (2)	0.0437 (19)	0.0432 (19)	0.0106 (16)	0.0036 (16)	−0.0111 (16)
C6	0.0138 (13)	0.0484 (19)	0.0434 (18)	−0.0013 (12)	−0.0026 (12)	−0.0089 (16)
C17	0.082 (3)	0.057 (2)	0.061 (3)	−0.014 (2)	−0.015 (2)	0.017 (2)

### Bis{5-(4-chlorophenyl)-3-[6-(1H-pyrazol-1-yl)pyridin-2-yl]-1H-1,2,4-triazol-1-ido}nickel(II) methanol disolvate Geometric parameters (Å, º)

**Table:**

Ni1—N4^i^	2.092 (2)	C4—C5	1.386 (3)
Ni1—N4	2.092 (2)	C8—C7	1.385 (3)
Ni1—N3	2.0383 (19)	C1—H1	0.9500
Ni1—N3^i^	2.0384 (19)	C1—C2	1.396 (4)
Ni1—N1	2.155 (2)	C5—H5	0.9500
Ni1—N1^i^	2.155 (2)	C5—C6	1.374 (4)
Cl1—C14	1.737 (3)	C12—H12	0.9500
N4—N5	1.363 (3)	C12—C13	1.386 (4)
N4—C9	1.344 (3)	C15—H15	0.9500
N3—C4	1.320 (3)	C15—C16	1.386 (4)
N3—C8	1.346 (3)	C15—C14	1.371 (4)
N6—C9	1.337 (3)	C16—H16	0.9500
N6—C10	1.355 (3)	C14—C13	1.377 (4)
O1—C17	1.396 (4)	C3—H3	0.9500
O1—H1A	0.84 (3)	C3—C2	1.363 (4)
N1—N2	1.378 (3)	C7—H7	0.9500
N1—C1	1.325 (3)	C7—C6	1.388 (4)
N2—C4	1.409 (3)	C2—H2	0.9500
N2—C3	1.352 (3)	C13—H13	0.9500
N5—C10	1.347 (3)	C6—H6	0.9500
C9—C8	1.458 (3)	C17—H17A	0.9800
C11—C10	1.470 (4)	C17—H17B	0.9800
C11—C12	1.386 (4)	C17—H17C	0.9800
C11—C16	1.392 (4)		
			
N4—Ni1—N4^i^	95.63 (11)	N3—C8—C9	111.8 (2)
N4^i^—Ni1—N1	91.57 (8)	N3—C8—C7	120.6 (2)
N4—Ni1—N1^i^	91.58 (8)	C7—C8—C9	127.5 (2)
N4—Ni1—N1	153.19 (7)	N1—C1—H1	124.3
N4^i^—Ni1—N1^i^	153.19 (7)	N1—C1—C2	111.4 (2)
N3—Ni1—N4	77.66 (8)	C2—C1—H1	124.3
N3^i^—Ni1—N4^i^	77.66 (8)	C4—C5—H5	121.8
N3^i^—Ni1—N4	105.57 (8)	C6—C5—C4	116.3 (2)
N3—Ni1—N4^i^	105.58 (8)	C6—C5—H5	121.8
N3—Ni1—N3^i^	175.32 (12)	C11—C12—H12	119.4
N3—Ni1—N1	75.52 (8)	C11—C12—C13	121.3 (3)
N3^i^—Ni1—N1	101.19 (8)	C13—C12—H12	119.4
N3—Ni1—N1^i^	101.19 (8)	C16—C15—H15	120.0
N3^i^—Ni1—N1^i^	75.52 (8)	C14—C15—H15	120.0
N1^i^—Ni1—N1	93.53 (11)	C14—C15—C16	119.9 (3)
N5—N4—Ni1	140.50 (15)	C11—C16—H16	119.8
C9—N4—Ni1	113.52 (16)	C15—C16—C11	120.3 (3)
C9—N4—N5	105.97 (19)	C15—C16—H16	119.8
C4—N3—Ni1	121.12 (16)	C15—C14—Cl1	119.8 (2)
C4—N3—C8	120.0 (2)	C15—C14—C13	120.9 (3)
C8—N3—Ni1	118.84 (16)	C13—C14—Cl1	119.3 (3)
C9—N6—C10	101.6 (2)	N2—C3—H3	126.7
C17—O1—H1A	108 (2)	N2—C3—C2	106.7 (2)
N2—N1—Ni1	112.27 (14)	C2—C3—H3	126.7
C1—N1—Ni1	143.26 (18)	C8—C7—H7	120.9
C1—N1—N2	104.4 (2)	C8—C7—C6	118.2 (2)
N1—N2—C4	117.7 (2)	C6—C7—H7	120.9
C3—N2—N1	111.6 (2)	C1—C2—H2	127.0
C3—N2—C4	130.6 (2)	C3—C2—C1	106.0 (2)
C10—N5—N4	105.23 (19)	C3—C2—H2	127.0
N4—C9—C8	118.0 (2)	C12—C13—H13	120.5
N6—C9—N4	113.8 (2)	C14—C13—C12	119.0 (3)
N6—C9—C8	128.1 (2)	C14—C13—H13	120.5
C12—C11—C10	119.6 (2)	C5—C6—C7	121.4 (2)
C12—C11—C16	118.5 (3)	C5—C6—H6	119.3
C16—C11—C10	121.9 (2)	C7—C6—H6	119.3
N6—C10—C11	122.4 (2)	O1—C17—H17A	109.5
N5—C10—N6	113.4 (2)	O1—C17—H17B	109.5
N5—C10—C11	124.1 (2)	O1—C17—H17C	109.5
N3—C4—N2	113.2 (2)	H17A—C17—H17B	109.5
N3—C4—C5	123.5 (2)	H17A—C17—H17C	109.5
C5—C4—N2	123.3 (2)	H17B—C17—H17C	109.5
			
Ni1—N4—N5—C10	−178.80 (19)	C9—N6—C10—N5	−1.1 (3)
Ni1—N4—C9—N6	178.29 (16)	C9—N6—C10—C11	176.9 (2)
Ni1—N4—C9—C8	−5.4 (3)	C9—C8—C7—C6	176.6 (3)
Ni1—N3—C4—N2	4.2 (3)	C11—C12—C13—C14	0.4 (5)
Ni1—N3—C4—C5	−175.7 (2)	C10—N6—C9—N4	1.4 (3)
Ni1—N3—C8—C9	−0.9 (3)	C10—N6—C9—C8	−174.6 (3)
Ni1—N3—C8—C7	176.8 (2)	C10—C11—C12—C13	−177.6 (3)
Ni1—N1—N2—C4	−2.1 (3)	C10—C11—C16—C15	177.0 (2)
Ni1—N1—N2—C3	−178.00 (17)	C4—N3—C8—C9	−178.1 (2)
Ni1—N1—C1—C2	176.5 (2)	C4—N3—C8—C7	−0.5 (4)
Cl1—C14—C13—C12	−179.6 (2)	C4—N2—C3—C2	−174.7 (3)
N4—N5—C10—N6	0.4 (3)	C4—C5—C6—C7	0.0 (4)
N4—N5—C10—C11	−177.5 (2)	C8—N3—C4—N2	−178.6 (2)
N4—C9—C8—N3	4.2 (3)	C8—N3—C4—C5	1.5 (4)
N4—C9—C8—C7	−173.3 (3)	C8—C7—C6—C5	0.9 (4)
N3—C4—C5—C6	−1.2 (4)	C1—N1—N2—C4	175.2 (2)
N3—C8—C7—C6	−0.7 (4)	C1—N1—N2—C3	−0.7 (3)
N6—C9—C8—N3	180.0 (2)	C12—C11—C10—N6	19.7 (4)
N6—C9—C8—C7	2.5 (4)	C12—C11—C10—N5	−162.5 (3)
N1—N2—C4—N3	−1.1 (3)	C12—C11—C16—C15	−0.6 (4)
N1—N2—C4—C5	178.9 (2)	C15—C14—C13—C12	−0.4 (5)
N1—N2—C3—C2	0.5 (3)	C16—C11—C10—N6	−157.9 (3)
N1—C1—C2—C3	−0.4 (3)	C16—C11—C10—N5	19.9 (4)
N2—N1—C1—C2	0.7 (3)	C16—C11—C12—C13	0.1 (4)
N2—C4—C5—C6	178.9 (2)	C16—C15—C14—Cl1	179.1 (2)
N2—C3—C2—C1	0.0 (3)	C16—C15—C14—C13	−0.1 (5)
N5—N4—C9—N6	−1.2 (3)	C14—C15—C16—C11	0.6 (4)
N5—N4—C9—C8	175.2 (2)	C3—N2—C4—N3	173.9 (2)
C9—N4—N5—C10	0.4 (3)	C3—N2—C4—C5	−6.2 (4)

Symmetry code: (i) −*x*+1, *y*, −*z*+3/2.

### Bis{5-(4-chlorophenyl)-3-[6-(1H-pyrazol-1-yl)pyridin-2-yl]-1H-1,2,4-triazol-1-ido}nickel(II) methanol disolvate Hydrogen-bond geometry (Å, º)

**Table:**

*D*—H···*A*	*D*—H	H···*A*	*D*···*A*	*D*—H···*A*
C2—H2···C14^ii^	0.95	2.86	3.73 (4)	153
C2—H2···C15^ii^	0.95	2.74	3.686 (4)	178
C2—H2···C16^ii^	0.95	2.88	3.743 (4)	151
C3—H3···O1^iii^	0.95	2.35	3.259 (4)	160
C5—H5···O1^iii^	0.95	2.47	3.399 (4)	167
C1—H1···N6^iv^	0.95	2.31	3.245 (6)	170
C7—H7···C1^iv^	0.95	2.70	3.611 (4)	161
O1—H1*A*···N5	0.84	1.96	2.795 (6)	176

Symmetry codes: (ii) −*x*+1, *y*+1, −*z*+3/2; (iii) *x*+1/2, *y*+1/2, −*z*+3/2; (iv) *x*−1/2, *y*+1/2, −*z*+3/2.
